# Investigating genetically stratified subgroups to better understand the etiology of alcohol misuse

**DOI:** 10.1038/s41380-023-02174-0

**Published:** 2023-07-25

**Authors:** Anaïs B. Thijssen, Spit for Science Working Group, Karen Chartier, Ananda Amstadter, Danielle M. Dick, Emily Lilley, Renolda Gelzinis, Anne Morris, Katie Bountress, Amy E. Adkins, Nathaniel Thomas, Zoe Neale, Kimberly Pedersen, Thomas Bannard, Seung B. Cho, Peter Barr, Holly Byers, Erin C. Berenz, Erin Caraway, James S. Clifford, Megan Cooke, Elizabeth Do, Alexis C. Edwards, Neeru Goyal, Laura M. Hack, Lisa J. Halberstadt, Sage Hawn, Sally Kuo, Emily Lasko, Jennifer Lend, Mackenzie Lind, Elizabeth Long, Alexandra Martelli, Jacquelyn L. Meyers, Kerry Mitchell, Ashlee Moore, Arden Moscati, Aashir Nasim, Jill Opalesky, Cassie Overstreet, A. Christian Pais, Tarah Raldiris, Jessica Salvatore, Jeanne Savage, Rebecca Smith, David Sosnowski, Jinni Su, Chloe Walker, Marcie Walsh, Teresa Willoughby, Madison Woodroof, Jia Yan, Cuie Sun, Brandon Wormley, Brien Riley, Fazil Aliev, Roseann Peterson, Bradley T. Webb, Danielle M. Dick, Danielle Posthuma, Jeanne E. Savage

**Affiliations:** 1 Department of Complex Trait Genetics, Center for Neurogenomics and Cognitive Research, Vrije Universiteit Amsterdam, Amsterdam Neuroscience, Amsterdam, The Netherlands; 2 Department of Psychiatry, Robert Wood Johnson Medical School, Rutgers—The State University of New Jersey, Piscataway, NJ USA; 3 Department of Clinical Genetics, Section Complex Trait Genetics, Amsterdam Neuroscience, Vrije Universiteit Medical Center, Amsterdam, The Netherlands; 4 Virginia Commonwealth University, Richmond, VA USA

**Keywords:** Addiction, Genetics, Psychology

## Abstract

Alcohol misuse (AM) is highly prevalent and harmful, with theorized subgroups differing on internalizing and externalizing dimensions. Despite known heterogeneity, genome-wide association studies (GWAS) are usually conducted on unidimensional phenotypes. These approaches have identified important genes related to AM but fail to capture a large part of the heritability, even with recent increases in sample sizes. This study aimed to address phenotypic heterogeneity in GWAS to aid gene finding and to uncover the etiology of different types of AM. Genetic and phenotypic data from 410,414 unrelated individuals of multiple ancestry groups (primarily European) in the UK Biobank were obtained. Mixture modeling was applied to measures of alcohol misuse and internalizing/externalizing psychopathology to uncover phenotypically homogenous subclasses, which were carried forward to GWAS and functional annotation. A four-class model emerged with “low risk”, “internalizing—light/non-drinkers”, “heavy alcohol use—low impairment”, and “broad high risk” classes. SNP heritability ranged from 3 to 18% and both known AM signals and novel signals were captured by genomic risk loci. Class comparisons showed distinct patterns of regional brain tissue enrichment and genetic correlations with internalizing and externalizing phenotypes. Despite some limitations, this study demonstrated the utility of genetic research on homogenous subclasses. Not only were novel genetic signals identified that might be used for follow-up studies, but addressing phenotypic heterogeneity allows for the discovery and investigation of differential genetic vulnerabilities in the development of AM, which is an important step towards the goal of personalized medicine.

## Introduction

Alcohol misuse (AM) comprises heavy alcohol consumption, binge drinking, and alcohol use disorder (AUD), which together cause significant financial and psychological burdens on individuals and on society [1]. The effectiveness of existing treatment and prevention programs is highly variable among individuals and predictions as to which participants will benefit from them are unreliable [2]. There is thus a critical need to discern the causes of individual differences in the development of AM and response to treatment.

Individuals likely differ in their neurobiological predispositions for developing an addiction, as is theorized by several long-standing typologies of alcohol misuse [3, 4]. Specifically, these typologies indicate different developmental etiologies of addiction for individuals with internalizing (mood/anxiety) versus externalizing (impulsivity/antisocial behavior) predispositions. Such typologies have also been demonstrated empirically [5–7] with mixture modeling approaches like latent class analysis (LCA). Mixture modeling reveals more homogenous “latent” subgroups based on similarity in patterns of response among observed variables. These subgroups are, in turn, more likely to have a homogenous etiology, making it easier for investigators to identify underlying causal connections. This technique could therefore be of great value for areas of research, like genetics, where etiology is particularly difficult to disentangle.

Despite the strong heritability (~50%) of both alcohol consumption [8] and AUD [9], identification of the underlying causal genes remains incomplete. Because complex phenotypes like AM are influenced by many genetic variants of small effect, the widespread assumption has been that the “missing heritability” problem would be solved by amassing larger sample sizes with enough power to detect variants of small effect. However, in the largest sample to date investigating alcohol consumption (*N* = 921 280), only 4.2% of the phenotypic variation was accounted for by genetic influences [10], a plateau in comparison to substantially smaller sample sizes (e.g., [11]). Insufficient sample sizes appear not to be the sole cause of the “missing heritability”.

A promising alternative strategy is to consider the presence of genetic heterogeneity [12], whereby distinct genetic pathways are involved for different subgroups of individuals or dimensions of AM. Accounting for genetic heterogeneity between AM phenotypes has already been shown to improve gene identification and interpretability of genetic results [13]. Considering heterogeneity between meaningfully distinct groups of individuals, such as the empirical subtypes identified by mixture models, could similarly improve our understanding of the genetic etiology of AM while having an even greater potential for direct application to personalized medicine. Further, the causal relationships between internalizing/externalizing psychopathology and AM subtypes are challenging to disentangle in observational research, but incorporating genetic tools like genetic correlation [14] and Mendelian randomization [15] could aid in resolving these etiological questions.

Several studies have already employed LCA to investigate phenotypic differences within AUD [5–7, 16], but few molecular genetic studies have followed suit. Studies investigating AM typically use a binary AUD diagnosis or a unidimensional alcohol-related measure (e.g., drinking quantity). These are straightforward approaches that can be easily implemented to gather large samples, but they fail to address phenotypic heterogeneity. The resulting sample will likely consist of many sub-phenotypes, making it challenging to detect genetic associations even in very large samples. Addressing the phenotypic heterogeneity of AM might therefore aid in uncovering more genetic signal, but, to date, only one study of AM has combined LCA with a genetic analysis [17]. This study identified three distinct classes based on patterns of AUD symptoms but was not able to detect replicable genetic variants associated with latent class membership, most likely because of the small sample size (*N* = 2 322).

In the current study, we investigated the genetic underpinnings of AM by taking into account the phenotypic heterogeneity of AM. We use mixture modeling to uncover different phenotypic classes in the large UK Biobank sample [18], and apply GWAS and in silico annotation tools to investigate the genetic etiology of these classes and their relationships to internalizing/externalizing phenotypes. This approach can improve understanding of the differential etiology of developing AM, thereby taking a step towards personalized medicine applications.

## Materials and methods

### Sample

Participants were volunteers of the UK Biobank (UKB), a population-based sample of ~500,000 adults in the UK aged 40–65 [18]. After providing informed consent, participants completed a self-report survey, and a subset (*n* = 157,366) later completed an online mental health questionnaire. Medical records of participants were linked via national health registries, and additional diagnoses were obtained through interviews and the online survey (self-reports of clinically diagnosed conditions). The National Research Ethics Service Committee North West–Haydock ethically approved this initiative (reference 11/NW/0382) and data were accessed under application #16406.

### Measures

#### Alcohol phenotypes

During the primary study visit, participants were surveyed about their drinking habits, including drinking status (current, former, lifetime abstainer), drinking patterns over the past 10 years (increase, decrease, stayed the same), typical drinking frequency (days per month), and typical drinking quantity (grams of ethanol per day, log transformed). Former drinkers and lifelong abstainers were excluded from frequency and quantity measures. The online assessment contained the AUDIT questionnaire [19], which includes questions about binge drinking and seven problems related to drinking, (e.g., guilt, concern from loved ones). ICD-10 diagnoses of AUD (code F10) or alcoholic cirrhosis (code K70) were derived from medical records/interviews. Lifelong abstainers were excluded from AUDIT and AUD measures. A full description of the measures can be found in Table ST1.

#### Internalizing phenotypes

Participants completed a neuroticism scale during the study visit and scales for recent anxiety and depression symptoms during the online mental health questionnaire. Lifetime diagnoses of major depressive disorder (MDD) and anxiety disorders (e.g., panic disorder [PD], specific phobias [SP], generalized anxiety disorder [GAD]) were derived from medical records and self-report (Table ST1).

#### Externalizing phenotypes

The follow-up questionnaire asked participants to self-report whether they had ever been addicted to any substance/behavior and about lifetime use of cannabis. ICD-10 diagnoses of tobacco or other substance use disorder (TUD, code F17; and SUD, codes F11–F16, F18, or F19) were obtained from medical records (Table ST1). Although drug use is only one facet of the externalizing spectrum, other measures such as impulsive personality traits were not collected in this sample.

### Data analysis

#### Mixture modeling

Mixture modeling was performed in Mplus version 8 [20] using a maximum likelihood estimation method with two through eight class models. All 24 items described above were included, and modeling was conducted on a subset of *n* = 410 961 unrelated individuals. Model selection was based on model entropy, posterior probabilities, and goodness-of-fit indices: Akaike’s information criterion (AIC) [21], Bayesian information criterion (BIC) [22], and sample-size–adjusted BIC (SSBIC) [23].

#### GWAS

Ancestry clustering and exclusions for relatedness and quality control (Supplementary Methods) resulted in a sample of 410,414 individuals from 5 ancestry groups: 387,013 European (EUR), 7831 African (AFR), 3511 from the Americas (AMR), 2411 East Asian (EAS), and 9648 South Asian (SAS). GWAS was performed separately for each ancestry group. Up to 16,977,415 single nucleotide polymorphisms (SNPs) were analyzed with PLINK [24], using a logistic regression model to predict membership between pairs of latent classes with age, sex, assessment center (EUR only), genotyping array, and 20 within-ancestry principal components (PCs) as covariates. Cross-ancestry results were meta-analyzed using METAL [25], weighted by sample size. However, since the non-EUR groups were very small (combined, ~5% of the total sample), we use the EUR-only results for follow-up analyses as these depend on ancestry-specific linkage disequilibrium (LD) and the other groups were underpowered to analyze individually. Two secondary EUR-only analyses were carried out, one including BMI as an extra covariate and one including SES as an extra covariate, given their known confounding effects on alcohol use [13, 26]. The genome-wide significance (GWS) threshold was *P* < 5 × 10^−8^. Follow-up in silico analyses were performed in FUMA [27] to determine genomic risk loci based on LD patterns of significant variants, ascertain the functional consequences of implicated variants, and test for enrichment of the GWAS association signal in genes/gene-sets (see Supplementary Methods for details).

#### Polygenic scores

To validate the GWAS results, we calculated polygenic scores (PGS) from the UKB latent class GWAS summary statistics in an independent sample in which a similar LCA was previously carried out [16]. Data came from “Spit for Science” (S4S; *n* = 7 666) [28, 29], a longitudinal study of genetic and environmental influences on mental health in students at a large, urban, public university in the U.S. Self-report measures were collected via the web-based REDCap system of electronic data capture tools [30] and used for LCA, resulting in three classes (“Low Risk”, “Internalizing”, and “Externalizing”). PRSice2 [31] was used to calculate PGS for S4S participants, then S4S class membership was predicted from their genetic liability for membership in the corresponding UKB latent class (Supplementary Methods).

#### Heritability and genetic correlation

Genome-wide SNP heritability and genetic correlations were computed using LD score regression (LDSC) [14] for the latent class GWAS summary statistics and nineteen publicly available GWAS summary statistics (Table ST12). GWASs were selected based on high quality and a phenotype related to either alcohol use (AUD diagnoses [32], AUDIT total score [33], typical [10] and maximum consumption [34], problematic alcohol use [32]), internalizing behavior/symptoms (anxiety [35], depressive symptoms [36], major depressive disorder [MDD] [37], neuroticism [38], subjective wellbeing [36]) or externalizing behavior/symptoms (age of smoking initiation [10], cannabis use disorder [CUD] [39], lifetime cannabis use [40], antisocial behavior [41], externalizing behavior [42, 43], risk tolerance [44], smoking initiation [10]). In addition, summary statistics for BMI and educational attainment were included because of their relationship with alcohol use [26] and socioeconomic status [45], respectively. See Supplementary Methods for additional details.

LAVA [46] was used to determine the local genetic overlap between AM phenotypes and latent class, beyond the global genome-wide genetic correlations estimated by LDSC. With this method we sought to determine whether specific regions of the genome previously linked to unidimensional measures of AM are also implicated in latent class membership. First, GWS alcohol-related risk loci were selected from previous large-scale GWAS [10, 32, 33, 47, 48] and 98 distinct alcohol-associated loci were defined (Supplementary Methods). Then, for each of these loci, the local genetic correlation was calculated between latent class membership and 3 genetically distinct alcohol-related dimensions: consumption [10], AUDIT total scores [33], and AUD diagnoses [32].

#### Mendelian randomization

We applied Generalized Summary-data based Mendelian Randomization (GSMR) [15] to infer plausible causal relationships between internalizing/externalizing psychopathology and subtypes of AM. This method utilizes summary-level GWAS data to indicate causal associations between a putative risk factor (exposure) and an outcome by using independent genome-wide significant SNPs as instrumental variables to index the (phenotypic) effect of the exposure on the outcome. HEIDI-outlier detection (*P* value threshold of 0.01) was used to filter genetic instruments that show clear pleiotropic effects on both the exposure phenotype and the outcome phenotype.

For this analysis we selected unique internalizing/externalizing phenotypes that showed significant genetic correlations (*r*_*g*_) with class membership, and used independent (*r*^2^ = <0.1), GWS lead SNPs associated with these phenotypes to estimate their likely causal effect on being a “case” in each latent class comparison. The analyses were also run in the opposite direction, with latent class membership predicting internalizing/externalizing phenotypes, to test for bidirectional or reverse causality. When fewer than 10 lead SNPs were GWS, we lowered the threshold for SNP selection to 5 × 10^−5^ to ensure sufficient instruments for analysis.

This method estimates a putative causal effect of the exposure on the outcome (*b*_*xy*_) as a function of the relationship between the SNPs’ effects on the exposure (*b*_*zx*_) and the SNPs’ effects on the outcome (*b*_*zy*_), given the assumption that the effect of non-pleiotropic SNPs on an exposure (*x*) should be related to their effect on the outcome (*y*) in an independent sample only via mediation through the phenotypic causal pathway (*b*_*xy*_). When there is a significant (Bonferroni corrected *p* < 0.05/80 = 6.25 × 10^−4^) effect after filtering out pleiotropic SNPs, there is evidence of a plausible causal effect of the exposure on the outcome, with the effect size (*b*_*xy*_) interpretable as the expected change in SDs of a quantitative outcome or log odds ratio of a case-control outcome. In the absence of a bidirectional effect, or when the effect size of one direction is much stronger than the other, this points to a plausible directional causal effect between exposure and outcome.

#### Cross-ancestry analyses and locus replication

Cross-ancestry analyses were performed for each class comparison to test for replication of SNP effects. GWS SNPs in the EUR GWAS were compared for sign concordance to the corresponding SNPs in the other ancestry GWASs. Significance was determined using a one-tailed exact binomial test of the proportion of concordant SNPs. Locus replication was tested using the risk loci determined by FUMA (see Supplementary Methods). For each genomic risk locus in the EUR data all SNPs were compared for sign concordance to the corresponding SNPs in the other ancestry groups. A locus was considered replicated if at least one SNP in the region was sign concordant and had a one-tailed *P* value smaller than 0.05 divided by the total number of lead SNPs, which represent the number of independent association signals.

## Results

### Mixture model

**Fig. 1:**
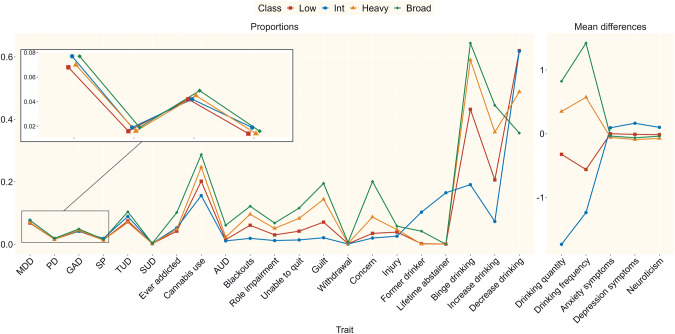
Patterns of endorsement of alcohol, internalizing, and externalizing items across the four latent classes. Probabilities and standardized mean differences are presented in separate panels. MDD Major depressive disorder. PD Panic disorder. GAD Generalized anxiety disorder. SP Specific Phobia. TUD Tobacco use disorder. SUD Substance use disorder. AUD Alcohol use disorder. Additional item descriptions can be found in Table ST1.

The prevalence of the variables in the model for the full sample are presented in Table ST1. The fit of two through eight class solutions were estimated (Table 1), and the four-class solution was chosen for its high entropy and because additional classes resulted in a plateauing of the improvement in model fit (Fig. SF1). Item endorsement probabilities of the classes are represented in Fig. 1 and demographic characteristics are shown in Table S2.

**Table 1:** Fit statistics from the mixture model.

Model	AIC	BIC	sBIC	Entropy	Post. Prob.
2-class	8704028.27	8704661.99	8704477.67	0.957	0.986
3-class	8587074.65	8588003.38	8587733.25	0.921	0.869
**4-class**	**7567653.29**	**7568877.03**	**7568521.09**	**0.980**	**0.989**
5-class	7519572.55	7521091.30	7520649.55	0.940	0.895
6-class	7478289.65	7480103.41	7479575.85	0.983	0.939
7-class	7437821.89	7439930.65	7439317.29	0.952	0.885
8-class	7268011.70	7270415.47	7269716.30	0.908	0.826

The bolded row represents the chosen solution.

*AIC* Akaike’s information criteria, *BIC* Bayesian information criteria, *sBIC* sample-size-adjusted Bayesian information criteria, *Post. Prob.* Average posterior probabilities of membership in assigned class.

Class 1 (“low risk”, “Low”, *n* = 105,142, 25.6%) was characterized by relatively low levels of alcohol consumption or problems and other disorders. Class 2 (“internalizing—light/non-drinkers”, “Int” *n* = 125,318, 30.5%) showed the lowest amount of consumption and alcohol problems (but many former drinkers and abstainers) as well as high scores on variables related to internalizing psychopathology. Class 3 (“heavy alcohol use—low impairment”, “Heavy”, *n* = 94,731, 23.1%) showed a relatively high endorsement of consumption and binge drinking, but without correspondingly high levels of AUDs or self-reports of addiction, and without elevated levels of most internalizing or externalizing problems. Class 4 (“broad high risk”, “Broad”, *n* = 85 770, 21.9%) had the highest levels of all alcohol items, AUDs, and of most internalizing and externalizing disorders.

### GWAS

Each class was compared pairwise to each other class, resulting in six GWASs per ancestry. Across all analyses, the lighter-drinking class served as the reference group for effect size estimation (i.e., for Int/Heavy/Broad vs. Low class comparisons, Low is always the reference group). As results did not differ substantively between the largest (~95% of the sample) EUR ancestry subgroup and the trans-ancestral meta-analysis (described later), follow-up analyses are based on the EUR-only GWAS.

**Fig. 2:**
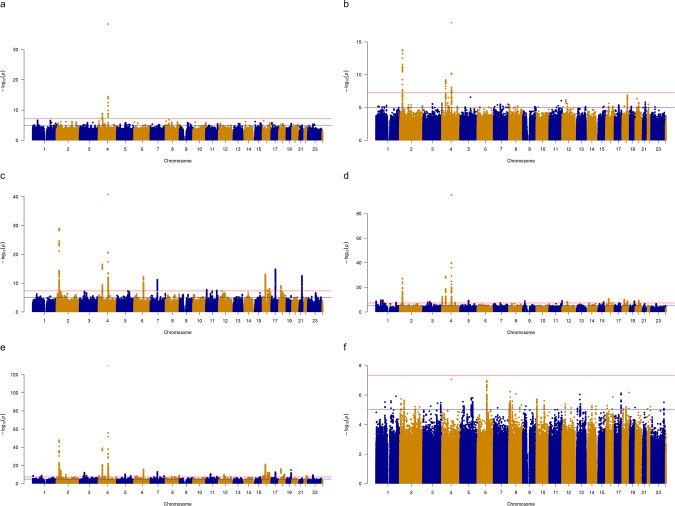
Manhattan plots for GWAS of latent class comparisons. Each GWAS illustrates a pairwise comparison between membership in the latent classes shown in Fig. 1: **a** Int vs. Low; **b** Heavy vs. Low; **c** Broad vs. Low; **d** Heavy vs. Int; **e** Broad vs. Int; **f** Broad vs. Heavy.

For the EUR GWASs, SNP-based heritability (on the liability scale) for the comparisons between classes ranged from 0.033 (s.e. 0.004) for Broad vs. Heavy to 0.183 (s.e. 0.008) for Broad vs. Int (Table ST3). Figure 2 shows the Manhattan plots and Fig. SF2 shows the QQ plots. Across analyses, a total of 96 genetic risk loci were found (Table ST4), of which 33 have not previously been associated with alcohol-related phenotypes [10, 11, 32, 33, 44, 47] and 6 were not associated with any phenotype in the NHGRI GWAS catalog (Table ST4, ST5, Figs. SF3–SF5). The loci also contained 22 novel exonic nonsynonymous (ExNS) SNPs not previously linked to alcohol-related phenotypes (Table ST6), which may have direct functional relevance. A total of 2214 candidate genes (Table ST7) were mapped to the risk loci, with the strongest associations found in the *ADH* and *KLB* gene regions that have been identified in numerous previous alcohol-related GWASs. The genetic signal was partially shared between classes, with 31/96 overlapping loci, 972/2214 overlapping implicated genes, and robust genetic correlations between nearly all class comparisons (Table ST3).

Genetic risk loci with GWS SNPs were found for all comparisons except Broad vs. Heavy classes (Table ST4). There were 2, 3, 16, 25, and 50 associated loci for the comparisons of Int-Low, Heavy-Low, Broad-Low, Heavy-Int, and Broad-Int, respectively. The number of identified loci increased in step with the degree of difference in alcohol consumption and problems between classes, with the strongest signal in the Broad-Int comparison. Significant SNPs in the Int-Low and Heavy-Low comparisons were linked to known genes related to alcohol consumption (*ADH1B, KLB, GCKR*), but novel alcohol-related loci and functional variants were identified for the other three comparisons (Supplementary Results). Some were significant across multiple class comparisons, such as a locus on 18q11 which contained the gene *NPC1* and multiple significant ExNSs. On the other hand, class-specific candidate genes from these novel loci included *MPHOSPH9* (Broad-Int), which is associated with expression differences from selective breeding of mice for alcohol preference [49] and PTSD symptoms [50], and *FAF1* (Heavy-Int), which mediates apoptosis. Of particular interest for follow-up were 15 novel loci that remained significant after controlling for potential confounding from BMI and SES and which have also not been linked to other psychiatric disorders/traits that might be indexed by the latent class structure (Table 2). The GWASs furthermore identified several novel ExNS SNPs (Table ST6) in known alcohol-associated genes, including the sulfation catalyst *SULT1A2* (Broad-Low, Broad-Int) and the taste receptor *TAS2R38* (Broad-Int). The strongest candidate genes from the GWASs were enriched for expression in several tissues, particularly brain, heart, muscle, and liver (Table ST8), and during late childhood (Broad-Low only; Table ST9). Candidate genes for all classes were overrepresented in gene-sets with known associations to body size and cognitive measures, while the Broad-Low and Broad-Int genes showed enrichment in gene-sets related to neuropsychiatric phenotypes like autism, schizophrenia, and neuroticism (Table ST10). More detailed descriptions of the associated loci and their implicated genes/gene-sets are provided in the Supplementary Results.

**Table 2:** Novel genomic loci associated with latent class membership.

Class	Locus	CHR	Start BP	End BP	Lead SNP	P	Nearest Gene	GWAS Catalog Associations
Broad-Low	16	21	40516308	40741846	21:40710198:T_TA	2.93E-13	*HMGN1*	Age of menarche, BMI, platelets
Heavy-Int	1	1	933790	1019180	1:962891:C_T	1.91E-09	*AGRN*	Blood protein levels
Heavy-Int	2	1	50839740	51506413	1:50984962:C_T	2.50E-10	*FAF1*	Baldness, carcinoma, cholesterol, headache, hippocampal tail volume, lung function, math ability, type 2 diabetes
Heavy-Int	15	9	20662649	20694595	9:20662649:G_T	1.22E-09	*FOCAD*	-
Heavy-Int	22	18	21075441	21165409	18:21156719:C_T	7.27E-11	*NPC1*	BMI, cholesterol, cognitive ability, educational attainment
Heavy-Int	25	20	35493755	35737816	20:35567830:G_T	4.94E-10	*SAMHD1*	Airway wall thickness
Heavy-Int / Broad-Int	8/10	3	71492037	71611630	3:71557945:A_C	2.84E-09	*FOXP1*	Autism, cancer, cognitive ability, educational attainment, lung function, nasal polyps, vitiligo
Broad-Int	2	1	93514059	93519168	1:93519168:T_TA	1.19E-09	*MTF2*	-
Broad-Int	8	3	48731450	50209053	3:49250007:C_T	8.38E-13	*IHO1*	Age at first birth, age at menarche blood pressure, BMI, bone density, cognitive ability, educational attainment, protein levels, sunburns
Broad-Int	19	5	145451731	145705669	5:145660413:T_TTTTA	2.95E-09	*RBM27*	Educational attainment, math ability
Broad-Int	24	7	141668403	141673345	7:141673345:C_G	3.67E-09	*MGAM, TAS2R38*	Bitter taste perception
Broad-Int	28	8	143475927	143534117	8:143534117:C_T	1.10E-09	*BAI1*	Math ability
Broad-Int	32	12	71298675	71394179	12:71363764:C_T	3.52E-08	*CTD-2021H9.1*	-
Broad-Int	50	X	55360035	56653185	X:56274351:A_G	4.79E-10	*KLF8*	Antigen levels, corpuscular volume

Novel loci are defined as those with no previous significant associations reported in the NCBI GWAS catalog for phenotypes related to alcohol use or internalizing or externalizing psychopathology. These loci remained significant after controlling for BMI and socioeconomic status. Full locus information is presented in Table ST4 and full GWAS catalog information is in Table ST5.

### Cross-ancestry analyses and locus replication results

For the GWASs of the other ancestry groups, Manhattan plots can be found in Figs. SF6–9 and heritability estimates can be found in Table ST3. Almost all of the identified risk loci were either also found in the EUR GWAS or were likely spurious because they were rare and had *P* values close to the GWS threshold. A notable exception was a locus on chromosome 12 which was found in all EAS comparisons involving class 2 (Int) and tags a functional variant in the *ALDH2* gene. This variant results in an inability to break down the toxic byproducts of ethanol and has been shown to have a major impact on the use of alcohol in East Asian populations [51]. Meta-analysis of the ancestry-specific GWAS summary statistics did not substantially change the results (Fig. SF10; Table ST15). See the Supplementary Results for a more comprehensive overview of the ancestry specific results and the meta-analysis.

To compare consistency of the results (in aggregate) across ancestry groups, we tested for sign concordance between GWS SNPs from the EUR GWAS and the same SNPs in the other ancestry-specific GWASs. We observed highly significant concordance for four out of five AFR, five of five AMR, two of five EAS, and four of five SAS comparisons (Table S16). The Broad-Heavy EUR GWAS did not have any GWS SNPs, therefore no cross-ancestry analyses were done for that class comparison. For the Int-Low class comparison replications there were only 23 GWS SNPs, so the interpretation of these results may not be very meaningful. Of the 96 EUR loci, 21 were replicated in AFR, 10 in AMR, 8 in EAS, and 13 in SAS (Table S16). Three loci were consistently replicated, namely locus 2 from the Int-Low class comparison, locus 3 from the Heavy-Low comparison, and locus 11 from the Heavy-Int comparison, which in all three cases is the locus containing *ADH1B*.

### Polygenic scores

PGS were used to predict latent class membership in the independent S4S sample (Table ST11). Specifically, GWAS of the heavy alcohol use classes (Heavy and Broad) compared to the Low risk class in UKB were used to predict membership of the Internalizing and Externalizing classes of S4S as compared to the Low Risk reference class. The UKB comparison of heavy alcohol use classes (Broad vs. Heavy) was also used to predict Internalizing versus Externalizing class in S4S. In EUR participants, the UKB Broad-Low PGS significantly predicted a higher likelihood of membership in the Internalizing (*R*^2^ = 0.6%, *P* = 0.00025), but not Externalizing class (*R*^2^ = 0.2%, *P* = 0.055). In EAS participants, the UKB Heavy-Low PGS significantly predicted a lower likelihood of membership in the Externalizing (*R*^2^ = 5.6%, *P* = 0.0005), but not Internalizing class (*R*^2^ = 0.8%, *P* = 0.063). No other predictions were significant after multiple testing correction.

### Genetic correlation

Genetic correlations between UKB latent classes and psychiatric traits/disorders can be found in Table ST12. Membership in the Heavy or Broad drinking classes was significantly correlated with higher typical alcohol consumption (*r*_*g*_ = 0.70–0.82), maximum consumption (*r*_*g*_ = 0.15–0.28), AUDIT score (*r*_*g*_ = 0.65–0.84), AUD risk (*r*_*g*_ = 0.27–0.44), and educational attainment (*r*_*g*_ = 0.39–0.49) and lower BMI (*r*_*g*_ = −0.26 to −0.32). Compared to other classes, membership in the Int class was correlated with higher depression and neuroticism (*r*_*g*_ = 0.10–0.43). Membership in the Broad class relative to the Heavy class was correlated with higher alcohol consumption/problems, alongside higher risk tolerance (*r*_*g*_ = 0.29), externalizing behavior (*r*_*g*_ = 0.27), cannabis use (*r*_*g*_ = 0.34) and likelihood of smoking (*r*_*g*_ = 0.24). Local genetic correlation analysis (Table ST13) indicated that the heritability of latent class membership was enriched in 35 out of 98 known alcohol-related loci. There were significant genetic correlations between membership in heavier alcohol-use classes and higher alcohol consumption/AUDIT/AUD at many of these loci, most strongly the *ADH* locus.

### Mendelian randomization

Using SNPs as instrumental variables in Mendelian randomization analysis, there was evidence of unidirectional and bidirectional causality between internalizing/externalizing phenotypes and latent class membership (Table ST14). Of particular interest, higher risk tolerance appeared to be a strong driver of membership in the Broad class as compared to Low (*b*_*xy*_ = 0.509, *P* = 4.0E-4), Int (*b*_*xy*_ = 0.674, *P* = 3.0E-6), and Heavy (*b*_*xy*_ = 0.588, *P* = 6.2E-5) classes, and being a smoker further distinguished the Broad from the Heavy class (*b*_*xy*_ = 0.092, *P* = 1.5E-4). Risk tolerance and externalizing behavior had a stronger effect on membership in the Broad versus Heavy class than vice versa, although there was evidence of bidirectionality.

## Discussion

In this study, we reduced the phenotypic heterogeneity of AM by using mixture modeling to derive phenotypically similar subgroups and investigated the genetic differences between these subgroups through GWASs and in silico analysis. This strategy not only replicated known AM loci, but also led to identification of novel genetic loci associated with AM subgroups, demonstrating the utility of this approach. There was substantial genetic overlap between the classes, and the strongest contributor to power to detect associated variants appeared to stem from the quantitative degree of difference in alcohol consumption/problems between classes. However, comparison of the classes revealed differences in heritability, genomic risk loci, involved genes, and genetic correlations, providing evidence that genetic differences between the classes contribute to the identified phenotypic differences.

Contrary to our expectation, the mixture model did not result in two groups of clearly delineated “internalizing” and “externalizing” drinkers, but rather two groups of heavy drinkers (class 3, “Heavy” and class 4, “Broad”) who differed on whether or not they experienced an array of clinically significant problems across the internalizing, externalizing, and alcohol misuse spectra. The null results of the GWAS comparing these two groups indicate that these classes were genetically different from the other classes but not from each other, suggesting that environmental factors might moderate the experience of psychiatric problems in the presence of similar individual genetic risk. The presence of an additional “internalizing” class with a high proportion of former (problem) drinkers may also indicate that the relevant internalizing/externalizing group comparisons are actually between the Int and Broad classes. This would be consistent with prior theories [3, 4] in which the internalizing subtype (here, class 2) often experiences more transient problems with alcohol. This is also consistent with the Mendelian randomization results which show putative causal effects of internalizing/externalizing problems on membership in the Broad vs. Int class. However, this point remains speculative as more detailed longitudinal data about lifelong patterns of alcohol use is needed to be able to draw such a conclusion.

The GWAS results largely captured differences in consumption, which were partially confounded by many of the same factors (BMI, SES) which complicate the interpretation of GWASs of unidimensional alcohol measures. Item-level analyses or factor mixture models may be better suited to deal with these persistent confounders. However, our analyses uncovered several interesting novel associations, including the *NPC1* gene, which codes for an intracellular cholesterol transporter and emerged though multiple class comparisons. A recent study found that genes involved in cholesterol homeostasis are downregulated upon alcohol exposure to iPSC derived neural cell cultures [52]. As these authors argued, cholesterol is a precursor for neuroactive steroids that act on GABA receptors and inhibiting synthesis of these steroids results in reduced sedation in response to alcohol [53], providing a possible link between cholesterol homeostasis and alcohol use. Another interesting finding is the *SULT1A2* gene, which is involved in the sulfation of alcohol and plays a smaller role in alcohol metabolism alongside the better-known *ADH* and *ALDH* gene products [54].

This study comes with a few limitations. The available data from the UKB is narrow with regards to externalizing traits and represents a limited time window. Alcohol misuse is a putative cause of other psychiatric problems [55], complicating the etiological investigation of AM in the context of a vulnerability for internalizing or externalizing behaviors. Furthermore, within this dataset, absence of a particular diagnosis does not necessarily mean absence of the disorder, since it is very well possible that participants suffer from a disorder but do not seek help or have not come into contact with medical professionals in a way that might have elicited a diagnosis. Another important limitation is that the UKB respondents differ from the general population on key characteristics related to health and lifestyle, including the consumption of alcohol [56], which limits generalizability. The non-EUR ancestry groups and the replication sample were also small, and novel results require additional validation. However, there are currently few large-scale studies that collect individual data at such a fine-grained resolution. Deep phenotyping needs to become a standard component of biobanks and genetic studies before we can conclusively determine the utility of this type of research.

By incorporating information from neurobiology into the diagnosis of addiction, efforts are underway to make the move towards treating patients within a personalized medicine framework. This study highlights the potential of using genetic information as a further step towards understanding the etiology of AM, by highlighting the genetic heterogeneity between subclasses of individuals with different patterns of AM. Investigation of these genetic differences can lead to a better understanding of the particular biological vulnerabilities of subgroups to develop AM, insights that might ultimately be used to advance personalized medicine.

### Supplementary information

Supplementary Note

Supplementary Tables

## Data Availability

Available on request.

## Data availability

Genome-wide summary statistics will be made publicly available via https://ctg.cncr.nl/software/summary_statistics/ upon publication. Raw data from this study are available to qualified researchers via UK Biobank (https://www.ukbiobank.ac.uk/) and dbGaP (phs001754.v4.p2).
