# Supplementary material for: Investigating genetically stratified subgroups to better understand the etiology of alcohol misuse
Source: Mol Psychiatry. 2023 Jul 25;28(10):4225–33. doi: 10.1038/s41380-023-02174-0 (PMC10827662; doi:10.1038/s41380-023-02174-0)
Supplement: Supplementary file 1 — Supplementary Note [file 41380_2023_2174_MOESM1_ESM.docx]

Supplementary Note for

**Investigating genetically stratified subgroups to better understand the etiology of alcohol misuse**

Anaïs B. Thijssen, M.Sc.^1^, Spit for Science Working Group, Danielle M. Dick, PhD^2^, Danielle Posthuma, PhD^1,3^, Jeanne E. Savage, PhD^1*^

^1^ Department of Complex Trait Genetics, Center for Neurogenomics and Cognitive Research, Vrije Universiteit Amsterdam, Amsterdam Neuroscience, Amsterdam, The Netherlands

^2^ Department of Psychiatry, Robert Wood Johnson Medical School, Rutgers – The State University of New Jersey, Piscataway, NJ, USA

^3^ Department of Clinical Genetics, Section Complex Trait Genetics, Amsterdam Neuroscience, Vrije Universiteit Medical Center, Amsterdam UMC, Amsterdam, The Netherlands

Table of Contents

[Supplementary Methods 3](#_Toc135666502)

[Genotyping, quality control and imputation 3](#_Toc135666503)

[Ancestry 3](#_Toc135666504)

[Genomic risk loci 4](#_Toc135666505)

[Functional mapping and annotation 4](#_Toc135666506)

[Polygenic scores 6](#_Toc135666507)

[Heritability and genetic correlation 7](#_Toc135666508)

[Supplementary Results 10](#_Toc135666509)

[EUR-only GWAS 10](#_Toc135666510)

[GWASs of other ancestries 14](#_Toc135666511)

[Supplementary Figures 17](#_Toc135666512)

[Supplementary Figure 1. Mixture model fit statistics supporting selection of the four-class model solution. 17](#_Toc135666513)

[Supplementary Figure 2. QQ plots from the EUR latent class comparison GWASs. 18](#_Toc135666514)

[Supplementary Figure 3. Regional plots of novel loci from the EUR Broad-Low class GWAS (1vs4). 19](#_Toc135666515)

[Supplementary Figure 4. Regional plots of novel loci from the EUR Heavy-Int class GWAS (2vs3). 20](#_Toc135666516)

[Supplementary Figure 5. Regional plots of novel loci from the EUR Broad-Int class GWAS (2vs4). 22](#_Toc135666517)

[Supplementary Figure 6. Manhattan plots for GWAS of latent class comparisons in the African (AFR) ancestry group. 24](#_Toc135666518)

[Supplementary Figure 7. Manhattan plots for GWAS of latent class comparisons in the Americas (AMR) ancestry group. 25](#_Toc135666519)

[Supplementary Figure 8. Manhattan plots for GWAS of latent class comparisons in the East Asian (EAS) ancestry group. 26](#_Toc135666520)

[Supplementary Figure 9. Manhattan plots for GWAS of latent class comparisons in the South Asian (SAS) ancestry group. 27](#_Toc135666521)

[Supplementary Figure 10. Manhattan plots for GWAS of latent class comparisons in the trans-ancestry meta-analysis. 28](#_Toc135666522)

[References 29](#_Toc135666523)

# Supplementary Methods

Genotyping, quality control and imputation
 Genotyping was done in 106 batches using the Affymetrix UK Biobank (UKB) Axiom array and the Affymetrix UK BiLEVE Axiom array which have a 95% marker overlap (1). The UKB team carried out quality control (QC) and imputation to the combined UK10K/1000 Genomes panel, of which the details can be found in their paper (1). Additional QC was performed locally to exclude single nucleotide polymorphisms (SNPs) with a low minor allele frequency (MAF; < 0.0001), high missingness (> 0.05) and low imputation quality (INFO score ≤ 0.9) which resulted in 16 977 415 available markers for genome-wide association (GWAS). Imputed variants were converted to hardcall format for analysis using a certainty threshold of 0.9 (2). Individuals were removed based on UKB-provided information on genomic relatedness (3^rd^ degree or closer), discordant sex, sex aneuploidy, missing phenotype or covariate data, and withdrawn consent.

## Ancestry

Participants were assigned to their closest matching ancestry group by projecting ancestry principal components from the 1000 Genomes reference populations onto the UKB genotypes and assigning individuals to their closes matching ancestral continental population based on Mahalanobis distance (as described in (2)). We included individuals from 5 continental ancestry groups: European (EUR), African (AFR), American (AMR), East Asian (EAS), and South Asian (SAS). Within-ancestry principal components were computed for each group based on a set of independent (*r^2^*<0.1) autosomal SNPs with MAF >0.01 and INFO=1 using FlashPCA2 (3). We examine ancestry-specific GWAS results as well as a trans-ancestral meta-analysis. However, given the small sizes of the non-EUR groups in this dataset (~5% of the total sample), we use the EUR-only GWAS results for locus definition, gene mapping, and most follow-up *in silico* analyses.

Genomic risk loci
 FUMA, an online platform for functional mapping of genetic variants (4), was used to identify genomic risk loci for the EUR-only latent class summary statistics. First, independent significant (IndSigSNPs) were distinguished, which are SNPs that are genome-wide significant (GWS; *P<*5x10^-8^) and are independent from each other (*r*^2^<0.6). From these, candidate SNPs were determined that are in linkage disequilibrium (LD) at *r*^2^>0.6, using a reference panel of 10 000 randomly selected UKB participants of European ancestry. IndSigSNPs that were independent from each other at *r*^2^<0.1 were defined as lead SNPs. Lead SNPs with LD blocks less than 250kb apart were merged to designate the associated genomic “risk” loci containing all candidate SNPs.

Functional mapping and annotation
 **Functional SNP annotation.** FUMA was used for functional annotation of the variants in the genomic risk loci. All candidate SNPs that had a *P* value of < 1x10^-5^ and a MAF of >0.0001 were selected for annotation. Functional consequences of these SNPs were derived from *in silico* databases: ANNOVAR categories (5), combined annotation-dependent depletion (CADD) scores (6), RegulomeDB scores (7), and chromatin state (8). ANNOVAR uses gene-based annotation to predict SNP function (categories include intronic, intergenic and exonic). CADD scores predict the deleteriousness of a SNP to the gene function, with scores >12.37 being potentially pathogenic. RegulomeDB score uses eQTL and chromatin information to estimate regulatory functions of SNPs, with lower scores indicating more potential regulatory functions. Minimum chromatin state was annotated across tissues to SNPs.
 **Gene mapping.** FUMA was then used to map candidate SNPs to genes using three strategies: Positional mapping, eQTL mapping, and chromatin interaction (CI) mapping. Positional mapping uses physical distance (within 10-kb windows) to map SNPs to genes. eQTL maps SNPs to genes when allelic variation is significantly associated with gene expression, using information from GTEx (9), Blood eQTL browser (10), and BIOS QTL browser (11). CI mapping uses Hi-C data (12) to detect physical interactions between SNPs and genes via three-dimensional chromatin folding. For CI mapping, the mapped genes were restricted to only include pairs where the candidate SNP was in a known regulatory region and the CI linked to the promotor region of the mapped gene. A false discovery rate (FDR) of 0.05 was applied to eQTL mapping and an FDR of 1x10^-5^ was applied to chromatin mapping.
 **Gene-based analysis**. MAGMA (13), as implemented in the FUMA platform, was used for genome-wide gene-based association analysis (GWGAS) of the EUR-only latent class summary statistics. Input for the GWGAS were SNP-based *P* values from the GWAS. SNPs were mapped to 19 725 protein-coding genes from the NCBI gene definitions. Gene association tests were performed, using a Bonferroni-corrected GWS threshold of *P*<.05/19 725=2.53x10^-6^.
 **Gene-set analysis**. GENE2FUNC from the FUMA platform was used to test the genes that were implicated by all four strategies (positional mapping, eQTL mapping, CI mapping and GWGAS) for enrichment in 3 types of databases: 1) curated gene-sets from MSigBD (14), WikiPathways (15), and the NHGRI GWAS catalog (16); 2) gene expression values from 54 tissues obtained from GTEx; and 3) gene expression values across 11 developmental stages (9). All gene types were selected for hypergeometric background gene-set testing, Ensemble version v92 was used and a minimum of two genes per gene-set was required. Bonferroni correction for multiple testing was applied within each gene-set category.

Polygenic scores

Results from a prior LCA in the dataset Spit for Science (S4S) (17) were used to test the generalizability of the UKB latent class GWAS with a polygenic score method. Briefly, a number of items relating to internalizing (depression, anxiety, neuroticism), externalizing (antisocial behavior, illicit substance use, impulsivity traits), and AM (binge drinking, AUD symptoms), were included in the prior S4S LCA. The resulting 3-class solution included a “Low Risk” class with low endorsement of all categories, an “Internalizing” class with high levels of internalizing and AUD symptoms but not binge drinking, and an “Externalizing” class with high levels of externalizing and AUD symptoms as well as binge drinking. After subsetting the sample to those with genotype data passing quality control filters, as previously described (18) , there were 7 666 participants available for PGS analysis (Low risk class: n=5 380; Internalizing class: n=1 460; Externalizing class: n=826). These participants were further subset by genetic continental ancestry groups (n=3 846 EUR, n=1 636 AFR, n=821 AMR, n=739 EAS, and n=624 SAS) in order to avoid confounding in the results due to population stratification. Within-ancestry principal components (PCs) were previously calculated within each of these groups (18).

To compare results across studies, we first identified the relevant latent class pairings between the UKB and S4S results. We considered the Low/Low Risk classes in both studies to be comparable, and the UKB Int class to be unique, since the most similar S4S Internalizing class had high levels of internalizing symptoms but also high levels of AUD symptoms and moderate binge drinking levels which were absent in the UKB Int class. We thus focused on the UKB Heavy and Broad classes as those carrying genetic risk relevant to the etiology of AM and used these two classes to genetically predict membership in the two S4S classes relevant to AM: Internalizing and Externalizing. Specifically, we calculated PGS based on the UKB Heavy-Low and Broad-Low GWAS summary statistics and used these to predict membership in the S4S Internalizing or Externalizing class versus the Low Risk class, respectively, in order to see whether the genetic influences on class membership could distinguish those with high versus low overall AM risk. Then, we calculated PGS based on the UKB Broad-Heavy GWAS and predicted membership in the S4S Internalizing versus Externalizing class, in order to determine whether the AM subtypes were themselves genetically distinguishable.

PGS were calculated from the UKB GWAS summary statistics using PRSice2 (19) to identify the optimal P-value threshold for inclusion of SNPs in the PGS by maximizing prediction in the validation sample. SNPs were first filtered on several quality control criteria (imputation INFO score > .8, MAF > .05, missingness < .025) and default settings were used in PRSice2 for further quality control steps such as LD clumping and removing SNPs with mismatching alleles. Latent class membership was predicted from the derived PGS using a logistic regression model with one class (typically Low Risk) as the reference group. All analyses were calculated separately within ancestry groups, using the ancestry-matched GWAS summary statistics from UKB, and the first 10 within-ancestry PCs were included as covariates. The PGS explained variance was calculated as the change in Nagelkerke’s R2 between a covariate-only model and a covariate + PGS model.

Heritability and genetic correlation

Genome-wide SNP heritability and genetic correlation values were computed using LD score regression (LDSC) (20) and nineteen publicly available summary statistics from GWASs (Table ST12). Age of smoking initiation and subjective wellbeing were sign-inversed, to facilitate interpretation across traits, as scoring higher on these traits implies lower externalizing and internalizing problems respectively. Pre-calculated LD scores from the 1000 Genomes European reference population were obtained from the software developers, and LD scores for the other ancestry groups were calculated locally using the respective 1000 Genomes reference data. SNP heritability was calculated on the latent class summary statistics for each individual ancestry group with their respective LD scores, using default program settings for quality control. Only HapMap3 SNPs were included for analysis. Observed SNP-heritability was transformed to the liability scale by assuming that the population prevalence of each class was equivalent to the class prevalence in the full sample, given that the true population prevalence of the latent classes is unknown. Due to low/unstable heritability estimates in the non-EUR ancestry groups, the EUR-only summary statistics were used to conduct genetic correlations in LDSC. Bonferroni-correction (*P*<0.05/6*16=5.2×10^-4^) was applied with regard to the number of phenotypes tested and the number of class comparisons. LDSC was used again to calculate genetic correlations between the EUR-only GWAS summary statistics of the latent class comparisons.

Local genetic correlation was carried out using LAVA (21) to determine the fine-grained genetic overlap between latent class membership and other more traditional measures of alcohol misuse. We selected GWS alcohol-related risk SNPs/loci reported in previous large-scale GWAS of unidimensional alcohol phenotypes, including 99 loci for consumption (22), 15 loci for AUDIT total scores (23), 11 SNPs for AUD diagnoses (24), 13 and 10 SNPs for AUDIT-C and AUD diagnoses, respectively (25), and 42 SNPs for problematic alcohol use (26). For studies that provided index/lead SNPs rather than LD-defined loci boundaries, we padded each SNP's chromosomal location with +/-125kb to define an associated locus. The 190 total loci were then combined across studies. Overlapping or nearby (within 250kb) regions were merged within and across studies, resulting in 98 distinct alcohol-associated loci (Supplementary Table ST13).

For each of these loci, we calculated the local genetic correlation between latent class membership (EUR-only GWAS results) and 3 alcohol misuse dimensions: consumption (22), AUDIT total scores (23), and AUD diagnoses (26). Correlations were only calculated if there was sufficient evidence of enrichment in SNP heritability (*P*>.0001) for both phenotypes within the given locus. The EUR 1000 Genomes samples were used as an LD reference panel, as distributed with the software. Sample overlap was provided where known (for the UKB summary statistics) or estimated from LDSC genetic correlation analysis (as above).

# Supplementary Results

EUR-only GWAS

**Class 2 versus 1 (Int vs. Low)**. GWAS results showed 23 genome-wide significant SNPs, indexed by 2 lead SNPs, spanning the previously identified *KLB* and *ADH* loci, which remained significant after controlling for BMI and SES (Table ST4). No genes were implicated by all four strategies.

**Class 3 versus 1 (Heavy vs. Low)**. GWAS results implicated 119 genome-wide significant SNPs, indexed by 4 lead SNPs, spanning 3 known genomic loci (Table ST4). These loci were the *GCKR*, *KLB* and *ADH* loci, and remained significant after controlling for BMI and SES. No genes were implicated by all four strategies.

**Class 4 versus 1 (Broad vs. Low)**. GWAS results showed 4 573 GWS SNPs, indexed by 19 lead SNPs, spanning 16 genomic loci (Table ST4), including the *GCKR*, *KLB*, *ADH*, *DRD2*, *SLC39A8* and *FTO* loci and three loci (Figure SF3) that have not been previously linked to alcohol phenotypes (22–29). Controlling for BMI resulted in four loci becoming nonsignificant, including the *SLC39A8* and *FTO* loci and one newly identified locus on chromosome 5 near *LINC00992* (locus 5). Controlling for SES did not change the genomic risk loci. GWAS furthermore identified eight new GWS ExNS SNPs, among which were rs1136703 (*P* = 4.4 x 10^-12^) in *SULT1A2* and rs1788799 (*P* = 2.2 x 10^-09^) in *NPC1*. The *SULT1A2* gene catalyzes the sulfation of hormones, neurotransmitters and drugs and has a role in the sulfation of ethanol in the liver and small intestine (30). The *NPC1* gene codes for a protein that transports low-density lipoproteins to late endosomal or lysosomal compartments where they are hydrolyzed and released as free cholesterol. The gene is associated with obesity (31) and mice lacking a copy of this gene show weight gain and insulin resistance (32).

A total of 39 candidate genes were implicated by all four strategies (Table ST7). Expression of the candidate genes was enriched in 11 tissues (Table ST8), seven of which were from the brain (most strongly the amygdala [*P=*1.69×10^-7^] and hippocampus [*P=*8.39×10^-7^]), while developmental stages of brain samples showed that expression was enriched during late childhood (Table ST9). These candidate genes were also overrepresented in sets of genes with known associations to cognitive ability, autism/schizophrenia, body size, cholesterol, inflammatory bowel disease, and alcohol use disorder, as well as gene-sets for biological processes including DNA repair and response to DNA damage (Table ST10).
 **Class 3 versus 2 (Heavy vs. Int)**. GWAS results showed 1 556 genome-wide significant SNPs, indexed by 35 lead SNPs, spanning 25 genomic loci (Table ST4). Eleven new alcohol-related loci were identified (Figure SF4), of which one (locus 12) was also identified as an internalizing locus (33), one (locus 18) as an externalizing locus (22,34) and one (locus 5) as both internalizing and externalizing (22,35,36). Five of the novel loci were retained after controlling for BMI and SES, including one which is located on chromosome 1 (locus 2) and spans the *FAF1* gene, which mediates apoptosis. Chronic high exposure to alcohol is an important risk factor for apoptosis-mediated cardiac dysfunction (37). Another novel locus (#22) on chromosome 18q11 contained one independent significant SNP (18:21087531:C_T) that tagged two novel ExNS SNPs in the *NPC1* gene (Table ST6), distinct from the *NPC1* ExNS SNP found in the 1vs4 comparison. These ExNS SNPs had CADD scores of 9.05 and 22.5, suggesting potential deleteriousness. The independent SNP was furthermore in high LD (*r*^2^=0.71-0.88) with multiple variants within the NPC1 gene that had an RDB score of 1f-1d, indicating potential regulatory features. Locus 22 was located near a region recently linked to alcohol consumption in the UKB sample (27), which was overlapped by the *NPC1* locus in the Broad-Low comparison.

A total of 29 genes were implicated by all four strategies (Table ST7). Four tissues were enriched for the genes that were mapped by all four strategies, which included the substantia nigra and the liver (Table ST8). These candidate genes were most strongly enriched in also gene-sets with known associations to cognitive ability, body size, cholesterol, and inflammatory bowel disease (Table ST10).
 **Class 4 versus 2 (Broad vs. Int)**. GWAS results identified 7 398 genome-wide significant SNPs, indexed by 64 lead SNPs, spanning 50 genomic loci (Table ST4). Comparison with recent alcohol-related GWASs identified 18 new alcohol-related loci (Figure SF5), two of which (locus 13 and 35) have been related to externalizing (22,27,38) and one (locus 47) to internalizing by other studies (35,39,40). After controlling for BMI and SES, 32 of the loci retained significance, 8 of which were newly identified. GWAS furthermore identified 19 new GWS ExNS SNPs (Table ST6), including rs1136703 in *SULT1A2* (*P* = 2.0x10^-17^), which was discussed above, and the three SNPs in the *NPC1* gene that were identified for Broad-Low and Heavy-Int.

One of the newly identified loci (locus 3) was on chromosome 2p24 and spans multiple genes. The lead SNP was located within an intron of the *KLHL29* gene, which is differentially expressed in alcoholic hepatitis (41) and associated with obesity (42) and cardiovascular disease (43). Another new discovery was the 12q24 locus containing *MPHOSPH9* (locus 33), which is associated with expression differences from selective breeding of mice for alcohol preference (44). Variants in this gene are related to PTSD symptoms (45) and diabetes (46). This region further contains the *SBNO1* gene, which codes for a nuclear-localized transcriptional regulator that might be important for brain function (47) and is linked to Notch signaling which has been proposed to be involved in neurogenesis and neuronal migration (48). Another newly identified locus (locus 45) was on chromosome 18q12 and has a chromatin interaction with *SYT4*, which is a synaptotagmin gene that is involved in retrograde signal transduction (49), is needed for dopamine release (50) and is differentially expressed in macaque hippocampi after binge drinking (51). This locus emerged previously in a genetic EEG study of families with alcohol problems (52). A chromatin interaction was found between this region and the *PIK3C3* gene, which is involved in autophagy (53). Autophagy has been shown to occur in the mouse liver to protect it from alcohol damage (54), where *PIK3C3* mRNA levels increase in response to alcohol (55).

A total of 91 genes were implicated by all four strategies (Table ST7) and showed enrichment for 15 tissues, including nine from the brain (Table S8), but no enrichment for developmental stage (Table S9). As in the previous class comparisons, the candidate genes were enriched in genes known to be associated with cognitive ability, body size measures, and cholesterol. Like the Broad-Low comparison, there was also enrichment for genes associated with psychiatric disorders like schizophrenia and alcohol use disorder, but the genes associated with Broad vs. Int class membership also showed more broad enrichment for additional neuropsychiatric phenotypes like neuroticism, mood instability, and sleep. There was also significant enrichment in a number of biological pathway gene-sets, including, among others, nucleotide binding, drug binding, and DNA repair.

**Class 4 versus 3 (Broad vs. Heavy).** For the Broad-Heavy class comparison, GWAS results showed no genome-wide significant SNPs (*P* < 5×10^-8^). Furthermore, GWGAS did not implicate any genes with significant aggregate association signal.
 **General results**. In total, 65 unique genetic risk loci were found. Genetic risk loci that were found in all class comparisons (except Broad-Heavy, which had no significant loci) were the ADH and KLB loci. Across analyses 2 214 genes were implicated, of which 69 genes were common to all class comparisons (except 3 versus 4, which did not show any significant genes) and 1 242 genes were unique to one specific class comparison.

## GWASs of other ancestries

Manhattan plots for the AFR-only GWAS (*n* = 7 831) are shown in Figure SF6. These analyses identified several GWS loci that were not found in the EUR GWAS, including for Int-Low (chr17:17,070,961, 1 SNP), Heavy-Low (chr16:71,375,921, 1 SNP), Broad-Low (chr6:133,079,381-133,136,798, 17 SNPs), and Broad-Int (chr1:238,631,889, 1 SNP; chrX:33,090,177, 1 SNP; chrX:131,730,667, 1 SNP). However, all of the variants in these regions were rare (MAF < .04) and had *P*-values close to the GWS threshold (minimum 2.55×10^-9^), so we consider these to be likely spurious results rather than robust ancestry-specific genetic signals.

Manhattan plots for the AMR-only GWAS (*n* = 3 511) are shown in Figure SF7. There were no GWS loci in this analysis.

Manhattan plots for the EAS-only GWAS (*n* = 2 411) are shown in Figure SF8. These analyses also identified several GWS loci that were not found in the EUR GWAS, including for Int-Low (chr12:111,367,244-112,817,783, 15 SNPs), Heavy-Int (chr12:112,110,489-112,574,616, 9 SNPs), and Broad-Int (chr12:111,414,461-112,817,783, 12 SNPs; chrX:104,754,552, 1 SNP; chrX:123,774,798, 1 SNP). The chromosome 12 locus was found across all comparisons involving class 2 (Int) and tags a functional variant in *ALDH2*, whose influence on alcohol use in East Asian populations via an inability to breakdown the toxic by-products of ethanol is well documented (56). The minor alleles of all SNPs in this locus were significantly associated (4.35×10^-8^ to 1.34×10^-15^) with a higher likelihood of membership in the light/non-drinking internalizing class 2.

Manhattan plots for the SAS-only GWAS (*n* = 9 648) are shown in Figure SF9. There was a single GWS SNP in the Heavy-Int comparison (chrX:50,975,027), and two GWS loci in the Broad-Int comparison (chr4:35,105,771-35,142,460, 10 SNPs; and chrX:136,597,344, 1 SNP). The two single-SNP loci on the X chromosome were both rare (MAF < .002) and likely spurious. SNPs in the chromosome 4 locus had a MAF of 0.18-0.21 and were located in an intergenic region.

Meta-analysis of the ancestry-specific GWAS summary statistics did not substantially change the results (Figure SF10; Table ST15), although a few differences between the EUR-only GWAS and the trans-ancestry meta-analyses are of note. There were a total of 26 GWS loci across the meta-analyses that were not significant in the EUR-only GWAS. Of these, 24 had a EUR *P*-value just above the GWS threshold (between *P=*7.6×10^-7^ and *P=*5.8×10^-8^), while the other two (Broad-Low locus #8 and Heavy-Int locus #19 near the *ALDH2* gene) contained variants that were not present in the EUR ancestry group.

The Int-Low meta-analysis had one additional GWS locus in an intronic region of the *GRXCR1* gene on chromosome 4. The Heavy-Low meta-analysis also had one additional GWS locus in an intronic region of the gene *TCF4* on chromosome 18, which was previously implicated in the EUR Heavy-Int and Broad-Int comparisons. In the Broad-Low meta-analysis, a locus previously implicated in the Broad-Int comparison was now significant (chr2: 45,136,742-45,175,289), and an additional new locus (chr6:133,039,516-133,241,274) was found. The lead SNP was located inside of the genes *RPS12* and *SNORA33*, and there have been no previously reported associations for this region in the GWAS catalog. In the Heavy-Int GWAS, one locus from the EUR GWAS (chr4:135,829,379-135,909,243, *P=*2.33×10^-8^) was no longer significant but 7 new loci were identified. These included the *ALDH2* locus (#19), 2 loci that were identified in the EUR Broad-Int GWAS (#4 and #30), one locus in the gene *CADM2* previously identified in GWASs of alcohol consumption as well as neuroticism and anxiety (#9), one locus (#16) near *MIR100HG* with a previous link to alcohol consumption (22), one locus in the gene *NPAS3* with no reported associations to alcohol or neuropsychiatric conditions (#20), and one locus (#26), also newly significant in the Broad-Int meta-analysis, near the gene *LINC01477* with no reported GWAS catalog associations. The Broad-Int meta-analysis resulted in one locus from the EUR GWAS (chr4:98,373,818-99,161,352) becoming merged with the nearby *ADH1B* locus and 16 additional loci being identified. These include five loci (#5, 15, 29, 37, and 48) previously linked to alcohol consumption (e.g., the *AUTS2* gene), five loci (#2, 10, 17, 31, and 44) with no previously associations to alcohol phenotypes but a variety of hits in the GWAS catalog related to body size and or cognitive functioning, one locus (#4) in the gene *BCAR3* with previous links to liver fibrosis and hematology, and five loci (#19, 39, 55, 58, and 62) with no previous reported phenotype associations in the GWAS catalog. Finally, in the Broad-Heavy meta-analyses, there was a GWS locus on chromosome 6 which was previously implicated in the EUR Broad-Low and Broad-Int GWASs.

# Supplementary Figures

## Supplementary Figure 1. Mixture model fit statistics supporting selection of the four-class model solution.

*Note: -2LL = -2*loglikelihood of the model; AIC = Akaike’s information criterion; BIC = Bayesian information criterion; ssBIC = sample-size adjusted BIC.*

## Supplementary Figure 2. QQ plots from the EUR latent class comparison GWASs.


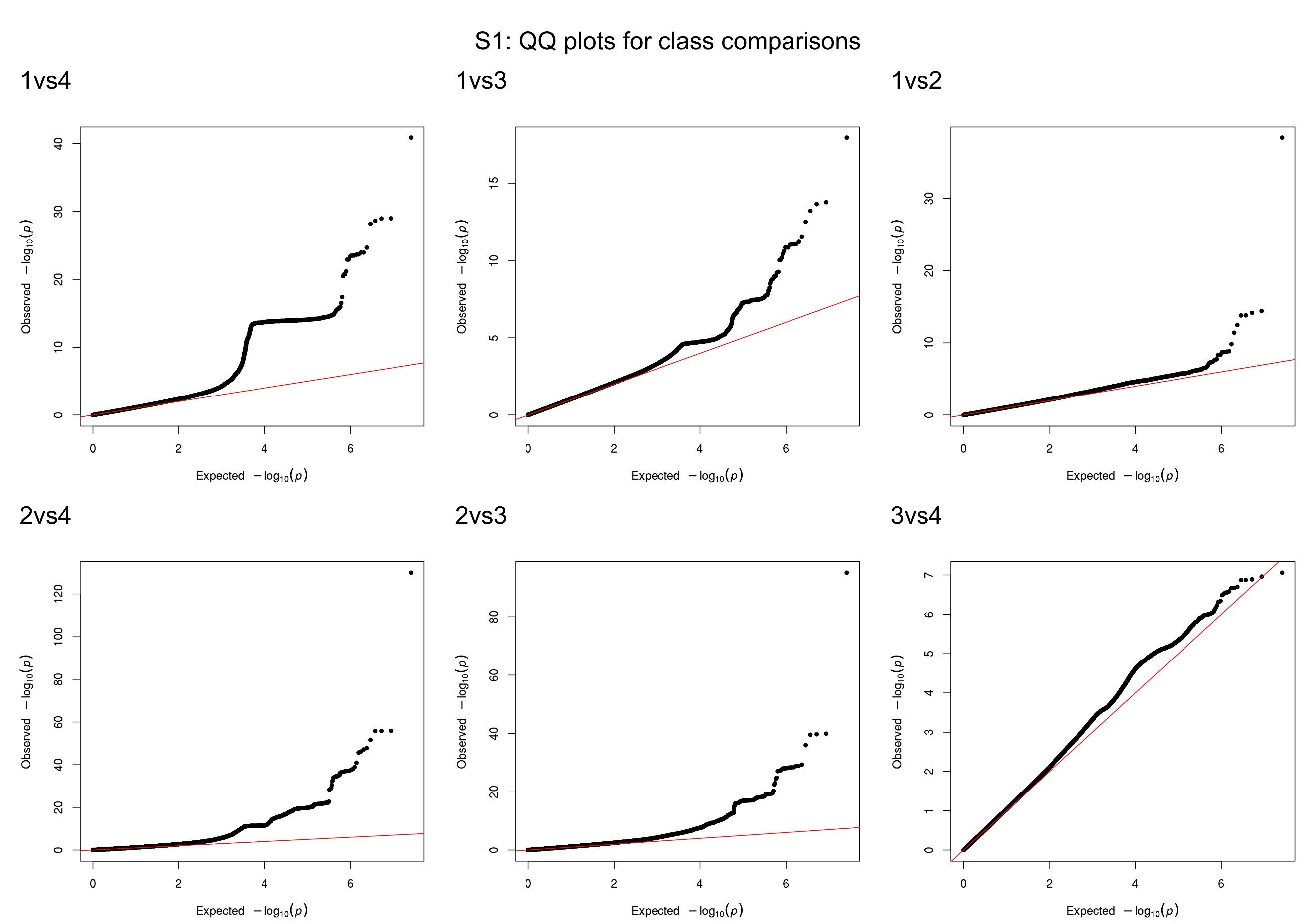


*Note: Inflation statistics are shown in Supplementary Table ST3. Class 1: “Low risk”; class 2: “Internalizing – light/non-drinkers”; class 3: “Heavy alcohol use – low impairment”; class 4: “Broad high risk”.*

## Supplementary Figure 3. Regional plots of novel loci from the EUR Broad-Low class GWAS (1vs4).

*Note: Novel loci are defined as those with no previous reported associations with alcohol consumption/problems phenotypes.*

## Supplementary Figure 4. Regional plots of novel loci from the EUR Heavy-Int class GWAS (2vs3).

*Note: Novel loci are defined as those with no previous reported associations with alcohol consumption/problems phenotypes.*

## Supplementary Figure 5. Regional plots of novel loci from the EUR Broad-Int class GWAS (2vs4).

*Note: Novel loci are defined as those with no previous reported associations with alcohol consumption/problems phenotypes.*

## Supplementary Figure 6. Manhattan plots for GWAS of latent class comparisons in the African (AFR) ancestry group.

*Note: Each GWAS illustrates a pairwise comparison between membership in the latent classes shown in Figure 1: a, Int vs. Low; b, Heavy vs. Low; c, Broad vs. Low; d, Heavy vs. Int; e, Broad vs. Int; f, Broad vs. Heavy.*

## Supplementary Figure 7. Manhattan plots for GWAS of latent class comparisons in the Americas (AMR) ancestry group.

*Note: Each GWAS illustrates a pairwise comparison between membership in the latent classes shown in Figure 1: a, Int vs. Low; b, Heavy vs. Low; c, Broad vs. Low; d, Heavy vs. Int; e, Broad vs. Int; f, Broad vs. Heavy.*

## Supplementary Figure 8. Manhattan plots for GWAS of latent class comparisons in the East Asian (EAS) ancestry group.

*Note: Each GWAS illustrates a pairwise comparison between membership in the latent classes shown in Figure 1: a, Int vs. Low; b, Heavy vs. Low; c, Broad vs. Low; d, Heavy vs. Int; e, Broad vs. Int; f, Broad vs. Heavy.*

## Supplementary Figure 9. Manhattan plots for GWAS of latent class comparisons in the South Asian (SAS) ancestry group.

*Note: Each GWAS illustrates a pairwise comparison between membership in the latent classes shown in Figure 1: a, Int vs. Low; b, Heavy vs. Low; c, Broad vs. Low; d, Heavy vs. Int; e, Broad vs. Int; f, Broad vs. Heavy.*

## Supplementary Figure 10. Manhattan plots for GWAS of latent class comparisons in the trans-ancestry meta-analysis.

*Note: Each GWAS illustrates a pairwise comparison between membership in the latent classes shown in Figure 1: a, Int vs. Low; b, Heavy vs. Low; c, Broad vs. Low; d, Heavy vs. Int; e, Broad vs. Int; f, Broad vs. Heavy.*

# References

1. Bycroft C, Freeman C, Petkova D, Band G, Elliott LT, Sharp K, e.a. The UK Biobank resource with deep phenotyping and genomic data. *Nature* 2018;**562**(7726):203-9.

2. Savage JE, Jansen PR, Stringer S, Watanabe K, Bryois J, de Leeuw CA, e.a. Genome-wide association meta-analysis in 269,867 individuals identifies new genetic and functional links to intelligence. *Nature Genetics* 2018;**50**(7):912-9.

3. Abraham G, Qiu Y, Inouye M. FlashPCA2: principal component analysis of Biobank-scale genotype datasets. Stegle O, redacteur. *Bioinformatics* 2017;**33**(17):2776-8.

4. Watanabe K, Taskesen E, van Bochoven A, Posthuma D. Functional mapping and annotation of genetic associations with FUMA. *Nature Communications* 2017;**8**(1):1826.

5. Wang K, Li M, Hakonarson H. ANNOVAR: functional annotation of genetic variants from high-throughput sequencing data. *Nucleic Acids Research* 2010;**38**(16):e164-e164.

6. Kircher M, Witten DM, Jain P, O’Roak BJ, Cooper GM, Shendure J. A general framework for estimating the relative pathogenicity of human genetic variants. *Nature Genetics* 2014;**46**(3):310-5.

7. Boyle AP, Hong EL, Hariharan M, Cheng Y, Schaub MA, Kasowski M, e.a. Annotation of functional variation in personal genomes using RegulomeDB. *Genome Res* 2012;**22**(9):1790-7.

8. Ernst J, Kellis M. ChromHMM: automating chromatin-state discovery and characterization. *Nature Methods* 2012;**9**(3):215-6.

9. Consortium TGte. The Genotype-Tissue Expression (GTEx) pilot analysis: Multitissue gene regulation in humans. *Science* 2015;**348**(6235):648-60.

10. Westra HJ, Peters MJ, Esko T, Yaghootkar H, Schurmann C, Kettunen J, e.a. Systematic identification of trans eQTLs as putative drivers of known disease associations. *Nature Genetics* 2013;**45**(10):1238-43.

11. Zhernakova DV, Deelen P, Vermaat M, van Iterson M, van Galen M, Arindrarto W, e.a. Identification of context-dependent expression quantitative trait loci in whole blood. *Nature Genetics* 2017;**49**(1):139-45.

12. Schmitt AD, Hu M, Jung I, Xu Z, Qiu Y, Tan CL, e.a. A Compendium of Chromatin Contact Maps Reveals Spatially Active Regions in the Human Genome. *Cell Reports* 2016;**17**(8):2042-59.

13. Leeuw CA de, Mooij JM, Heskes T, Posthuma D. MAGMA: Generalized Gene-Set Analysis of GWAS Data. *PLOS Computational Biology* 2015;**11**(4):e1004219.

14. Zhu Z, Zheng Z, Zhang F, Wu Y, Trzaskowski M, Maier R, e.a. Causal associations between risk factors and common diseases inferred from GWAS summary data. *Nature Communications* 2018;**9**(1):224.

15. Kutmon M, Riutta A, Nunes N, Hanspers K, Willighagen EL, Bohler A, e.a. WikiPathways: capturing the full diversity of pathway knowledge. *Nucleic Acids Res* 2016;**44**(D1):D488-94.

16. MacArthur J, Bowler E, Cerezo M, Gil L, Hall P, Hastings E, e.a. The new NHGRI-EBI Catalog of published genome-wide association studies (GWAS Catalog). *Nucleic Acids Res* 2017;**45**(D1):D896-901.

17. Savage JE, Spit for Science Working Group, Dick DM. Internalizing and externalizing subtypes of alcohol misuse and their relation to drinking motives. *Addict Behav* 2023;136:107461.

18. Peterson RE, Edwards AC, Bacanu SA, Dick DM, Kendler KS, Webb BT. The utility of empirically assigning ancestry groups in cross-population genetic studies of addiction. *The American Journal on Addictions*. 2017;**26**(5):494-501.

19. Choi SW, O’Reilly PF. PRSice-2: Polygenic Risk Score software for biobank-scale data. *GigaScience* 2019;**8**(7):giz082.

20. Bulik-Sullivan BK, Loh PR, Finucane HK, Ripke S, Yang J, Patterson N, e.a. LD Score regression distinguishes confounding from polygenicity in genome-wide association studies. *Nature Genetics* 2015;**47**(3):291-5.

21. Werme J, van der Sluis S, Posthuma D, de Leeuw CA. An integrated framework for local genetic correlation analysis. *Nat Genet* 2022;**54**(3):274-82.

22. Liu M, Jiang Y, Wedow R, Li Y, Brazel DM, Chen F, e.a. Association studies of up to 1.2 million individuals yield new insights into the genetic etiology of tobacco and alcohol use. *Nature Genetics* 2019;**51**(2):237-44.

23. Sanchez-Roige S, Palmer AA, Fontanillas P, Elson SL, Adams MJ, Howard DM, e.a. Genome-Wide Association Study Meta-Analysis of the Alcohol Use Disorders Identification Test (AUDIT) in Two Population-Based Cohorts. *AJP* 2018;**176**(2):107-18.

24. Walters RK, Polimanti R, Johnson EC, McClintick JN, Adams MJ, Adkins AE, e.a. Transancestral GWAS of alcohol dependence reveals common genetic underpinnings with psychiatric disorders. *Nature Neuroscience* 2018;**21**(12):1656-69.

25. Kranzler HR, Zhou H, Kember RL, Vickers Smith R, Justice AC, Damrauer S, e.a. Genome-wide association study of alcohol consumption and use disorder in 274,424 individuals from multiple populations. *Nature Communications* 2019;**10**(1):1499.

26. Zhou H, Sealock JM, Sanchez-Roige S, Clarke TK, Levey DF, Cheng Z, e.a. Genome-wide meta-analysis of problematic alcohol use in 435,563 individuals yields insights into biology and relationships with other traits. *Nature Neuroscience* 2020;**23**(7):809-18.

27. Karlsson Linnér R, Biroli P, Kong E, Meddens SFW, Wedow R, Fontana MA, e.a. Genome-wide association analyses of risk tolerance and risky behaviors in over 1 million individuals identify hundreds of loci and shared genetic influences. *Nat Genet* 2019;**51**(2):245-57.

28. Clarke TK, Adams MJ, Davies G, Howard DM, Hall LS, Padmanabhan S, e.a. Genome-wide association study of alcohol consumption and genetic overlap with other health-related traits in UK Biobank ( N =112 117). *Molecular Psychiatry* 2017;**22**(10):1376-84.

29. Evangelou E, Gao H, Chu C, Ntritsos G, Blakeley P, Butts AR, e.a. New alcohol-related genes suggest shared genetic mechanisms with neuropsychiatric disorders. *Nat Hum Behav* 2019;**3**(9):950-61.

30. Kurogi K, Davidson G, Mohammed YI, Williams FE, Liu MY, Sakakibara Y, e.a. Ethanol Sulfation by the Human Cytosolic Sulfotransferases: A Systematic Analysis. *Biological and Pharmaceutical Bulletin*. 2012;**35**(12):2180-5.

31. Bambace C, Dahlman I, Arner P, Kulyté A. NPC1 in human white adipose tissue and obesity. *BMC Endocrine Disorders* 2013;**13**(1):5.

32. Jelinek D, Millward V, Birdi A, Trouard TP, Heidenreich RA, Garver WS. Npc1 haploinsufficiency promotes weight gain and metabolic features associated with insulin resistance. *Human Molecular Genetics* 2011;**20**(2):312-21.

33. Okbay A, Baselmans BML, De Neve JE, Turley P, Nivard MG, Fontana MA, e.a. Genetic variants associated with subjective well-being, depressive symptoms, and neuroticism identified through genome-wide analyses. *Nat Genet* 2016;**48**(6):624-33.

34. David SP, Hamidovic A, Chen GK, Bergen AW, Wessel J, Kasberger JL, e.a. Genome-wide meta-analyses of smoking behaviors in African Americans. *Transl Psychiatry* 2012;2:e119.

35. Nagel M, Jansen PR, Stringer S, Watanabe K, de Leeuw CA, Bryois J, e.a. Meta-analysis of genome-wide association studies for neuroticism in 449,484 individuals identifies novel genetic loci and pathways. *Nature Genetics* 2018;**50**(7):920-7.

36. Turley P, Walters RK, Maghzian O, Okbay A, Lee JJ, Fontana MA, e.a. Multi-trait analysis of genome-wide association summary statistics using MTAG. *Nat Genet* 2018;**50**(2):229-37.

37. Fernández-Solà J, Fatjó F, Sacanella E, Estruch R, Bosch X, Urbano-Márquez A, e.a. Evidence of apoptosis in alcoholic cardiomyopathy. *Human Pathology* 2006;**37**(8):1100-10.

38. Martin J, Walters RK, Demontis D, Mattheisen M, Lee SH, Robinson E, e.a. A Genetic Investigation of Sex Bias in the Prevalence of Attention-Deficit/Hyperactivity Disorder. *Biol Psychiatry* 2018;**83**(12):1044-53.

39. Nagel M, Watanabe K, Stringer S, Posthuma D, van der Sluis S. Item-level analyses reveal genetic heterogeneity in neuroticism. *Nat Commun* 2018;**9**(1):905.

40. Hill WD, Weiss A, Liewald DC, Davies G, Porteous DJ, Hayward C, e.a. Genetic contributions to two special factors of neuroticism are associated with affluence, higher intelligence, better health, and longer life. *Mol Psychiatry* 2020;**25**(11):3034-52.

41. Yao J, Cheng Y, Zhang D, Fan J, Zhao Z, Li Y, e.a. Identification of key genes, MicroRNAs and potentially regulated pathways in alcoholic hepatitis by integrative analysis. *Gene* 2019;720:144035.

42. Comuzzie AG, Cole SA, Laston SL, Voruganti VS, Haack K, Gibbs RA, e.a. Novel Genetic Loci Identified for the Pathophysiology of Childhood Obesity in the Hispanic Population. *PLOS ONE* 2012;**7**(12):e51954.

43. Chan KHK, Huang YT, Meng Q, Wu C, Reiner A, Sobel EM, e.a. Shared Molecular Pathways and Gene Networks for Cardiovascular Disease and Type 2 Diabetes Mellitus in Women Across Diverse Ethnicities. Circulation: *Cardiovascular Genetics* 2014;**7**(6):911-9.

44. Hoffman PL, Saba LM, Flink S, Grahame NJ, Kechris K, Tabakoff B. Genetics of gene expression characterizes response to selective breeding for alcohol preference. *Genes, Brain and Behavior* 2014;**13**(8):743-57.

45. Sheerin CM, Kovalchick LV, Overstreet C, Rappaport LM, Williamson V, Vladimirov V, e.a. Genetic and Environmental Predictors of Adolescent PTSD Symptom Trajectories Following a Natural Disaster. *Brain Sciences* 2019;**9**(6):146.

46. Han X, Wei Y, Hu H, Wang J, Li Z, Wang F, e.a. Genetic Risk, a Healthy Lifestyle, and Type 2 Diabetes: the Dongfeng-Tongji Cohort Study. *The Journal of Clinical Endocrinology & Metabolism* 2020;**105**(4):1242-50.

47. Alberry BLJ, Castellani CA, Singh SM. Hippocampal transcriptome analysis following maternal separation implicates altered RNA processing in a mouse model of fetal alcohol spectrum disorder. *J Neurodevelop Disord* 2020;**12**(1):15.

48. Grill M, Syme TE, Noçon AL, Lu AZX, Hancock D, Rose-John S, e.a. Strawberry notch homolog 2 is a novel inflammatory response factor predominantly but not exclusively expressed by astrocytes in the central nervous system. *Glia* 2015;**63**(10):1738-52.

49. Yoshihara M, Adolfsen B, Galle KT, Littleton JT. Retrograde Signaling by Syt 4 Induces Presynaptic Release and Synapse-Specific Growth. *Science* 2005;**310**(5749):858-63.

50. Mendez JA, Bourque MJ, Fasano C, Kortleven C, Trudeau LE. Somatodendritic Dopamine Release Requires Synaptotagmin 4 and 7 and the Participation of Voltage-gated Calcium Channels*. *Journal of Biological Chemistry* 2011;**286**(27):23928-37.

51. Maxi JK, Dean M, Zabaleta J, Reiss K, Bagby GJ, Nelson S, e.a. Chronic Binge Alcohol Administration Dysregulates Hippocampal Genes Involved in Immunity and Neurogenesis in Simian Immunodeficiency Virus-Infected Macaques. *Biomolecules* 2016;**6**(4):43.

52. Yu Y, Meng Y, Ma Q, Farrell J, Farrer LA, Wilcox MA. Whole-genome variance components linkage analysis using single-nucleotide polymorphisms versus microsatellites on quantitative traits of derived phenotypes from factor analysis of electroencephalogram waves. *BMC Genetics* 2005;**6**(1):S15.

53. Yang G, Song W, Postoak JL, Chen J, Martinez J, Zhang J, e.a. Autophagy-related protein PIK3C3/VPS34 controls T cell metabolism and function. *Autophagy* 2021;**17**(5):1193-204.

54. Ding W, Li M, Chen X, Ni H, Lin C, Gao W, e.a. Autophagy Reduces Acute Ethanol-Induced Hepatotoxicity and Steatosis in Mice. *Gastroenterology* 2010;**139**(5):1740-52.

55. Ni HM, Du K, You M, Ding WX. Critical Role of FoxO3a in Alcohol-Induced Autophagy and Hepatotoxicity. *The American Journal of Pathology* 2013;**183**(6):1815-25.

56. Baik I, Cho NH, Kim SH, Han BG, Shin C. Genome-wide association studies identify genetic loci related to alcohol consumption in Korean men. *Am J Clin Nutr* 2011;**93**(4):809-16.
